# Prognostic Value of NT-proBNP in Patients Treated With Allogeneic Stem Cell Transplantation

**DOI:** 10.1016/j.jacadv.2025.102415

**Published:** 2025-12-11

**Authors:** Elissa A.S. Polomski, Peter A. von dem Borne, Hendrik Veelken, Julius C. Heemelaar, J. Wouter Jukema, M. Louisa Antoni

**Affiliations:** a Department of Cardiology, Heart Lung Center, Leiden University Medical Center, Leiden, the Netherlands; b Department of Hematology, Leiden University Medical Center, Leiden, the Netherlands; c Netherlands Heart Institute, Utrecht, the Netherlands

**Keywords:** allogeneic stem cell transplantation, cardiac biomarkers, cardio-oncology, NT-proBNP

## Abstract

**Background:**

As survival rates after allogeneic stem cell transplantation (alloSCT) continue to grow, cardiovascular disease is becoming a major complication after alloSCT. However, the prognostic value of cardiac biomarkers has not been widely investigated.

**Objectives:**

This study aims to investigate the association between baseline N-terminal prohormone of brain natriuretic peptide (NT-proBNP) in patients treated with alloSCT and mortality.

**Methods:**

Patients who were referred for alloSCT between 2012 and 2023 were included in this single-center retrospective cohort study if NT-proBNP was measured <180 days before alloSCT. The primary study outcome was all-cause mortality and the secondary study outcome was a composite endpoint of major adverse cardiac events (acute coronary syndrome, device implantation, arrhythmias, heart failure, and pericarditis).

**Results:**

We included 807 patients, of whom 34.5% were female with a median age of 58.6 (47.9-66.0) years who had a median NT-proBNP at baseline of 113.2 (56.9-229.4) ng/L. During follow-up of 1.6 (0.6-4.0) years, 393 patients (48.7%) died, of whom 61.8% due to a complication of alloSCT. Patients who died had a significant higher baseline NT-proBNP compared to survivors (122.4 [66.4-273.6] ng/L vs 100.2 [51.2-214.2] ng/L, *P* = 0.001). Patients with NT-proBNP ≥125 ng/L showed a lower 5-year survival rate (*P* = 0.0035). In a multivariable Cox regression model, log-transformed NT-proBNP (HR: 1.21 [95% CI: 1.11-1.31], *P* < 0.001) and NT-proBNP ≥125 ng/L (HR: 1.29 [95% CI: 1.04-1.60], *P* = 0.020) were positively associated with increased risk of death.

**Conclusions:**

Baseline NT-proBNP in patients treated with alloSCT is positively associated with an increased risk of all-cause mortality. NT-proBNP ≥125 ng/L was associated with a significant lower 5-year survival.

Allogeneic stem cell transplantation (alloSCT) is performed as treatment in various types of cancers and blood disorders, including lymphoma, myeloproliferative diseases, and myelodysplastic syndromes, with acute leukemia being the most common indication for alloSCT in Europe.^1^ Due to advances and improvements in alloSCT over the past years, survival has increased, and higher-risk patients (older age, advanced disease, and comorbidities) have become more eligible for this treatment.

As the number of survivors following alloSCT continues to grow, the likelihood of experiencing treatment-related complications, including cardiovascular disease (CVD), is increasing and becoming one of the most prevalent complications after alloSCT.^2^ Therefore, various studies have focused on the risk of CVD and cardiac events after alloSCT and observed that long-term survivors have increased risks of CVD, cardiovascular risk factors and death, in particular in the presence of pre-existing cardiovascular risk factors and events or prior treatment with anthracyclines.^3^, ^4^, ^5^, ^6^ The most common cardiovascular complications of alloSCT include arrhythmias and heart failure (HF).^7^^,^^8^ Current guidelines advise to perform baseline cardiovascular risk assessment, an electrocardiogram, echocardiogram (Class I), and measure natriuretic peptides (NPs) (Class IIa) for risk stratification and to detect subclinical cardiotoxicity from prior cancer treatments. Re-evaluation with these examinations is recommended in high-risk patients (pre-existing CVD, uncontrolled cardiovascular risk factors, graft-versus-host disease (GVHD), alloSCT or prior treatment with thoracic radiotherapy, high-dose anthracyclines, total body irradiation (TBI), or alkylating agents) after 2 and 12 months and in low-risk patients only if new cardiac symptoms develop.^8^^,^^9^

Prior research has investigated the progression of B-type natriuretic peptide (BNP) levels during several weeks after alloSCT. In all patients who developed HF, elevated BNP plasma levels (>43 pmol/L) were observed, supporting the use of NPs as a marker of ventricular dysfunction.^10^ This raises the question whether a baseline echocardiogram should be performed in all patients or should be reserved for high-risk patients, with low-risk patients being screened initially using only cardiac biomarkers. However, studies examining the possible prognostic value of baseline N-terminal prohormone of brain natriuretic peptide (NT-proBNP) on mortality are lacking. In 2012, measurements of pro-BNP concentrations were introduced as part of routine eligibility examinations immediately prior to alloSCT in our center. Based on this policy, this study aims to investigate the association between baseline NT-proBNP in patients treated with alloSCT and mortality.

## Methods

### Patient data

This single-center retrospective cohort study was conducted in patients treated with alloSCT. Patients referred to Leiden University Medical Center for alloSCT between 2012 and 2023 were screened for eligibility. Patients were included if NT-proBNP levels were measured prior to alloSCT and excluded if baseline NT-proBNP measurements were taken more than 180 days before the procedure. The cutoff for elevated NT-proBNP was ≥125 ng/L based on the European Society of Cardiology (ESC) guidelines on Cardio-Oncology and the Universal Definition of Heart Failure.^9^^,^^11^ The reference value for NT-proBNP of our laboratory was <161 ng/L. We defined reference values for normal NT-proBNP as <240 ng/L (females) and <195 ng/L (males) according to the median age of our cohort.^11^ Medication at baseline was defined as medication that was prescribed <1 year before alloSCT. Hypertension, hypercholesterolemia, and diabetes mellitus were all defined based on a documented medical history of these conditions and smoking was defined as current or prior smoking. We defined a complication of alloSCT as the cause of death if it was attributed to either GVHD, neutropenic sepsis, or autoimmune disease. This would be a better translation: The study was approved by the local non-Medical Research Involving Human Subjects Act (nWMO) committee (2023-061) and complies with the Declaration of Helsinki.

### Data collection

Patient demographics, medical history, medication, laboratory results, and oncological diagnosis and treatment were gathered from the institutional electronic patient chart and pharmacy records (HiX Version 6.3 Chipsoft). C-reactive protein, cholesterol levels, and triglycerides were collected if available. Data on cardiovascular history, risk factors, and events were collected through the departmental information system (EPD-Vision Version 12.14.0.1). The cause of death and date of last follow-up were obtained by manual chart review.

### Study outcomes

The primary study outcome was all-cause mortality and the secondary study outcome was a composite endpoint of major adverse cardiac events that are associated with alloSCT, defined as acute coronary syndrome (ACS) (ST-segment elevation myocardial infarction, non–ST-segment elevation myocardial infarction, unstable angina pectoris), device implantation, arrhythmias (supraventricular or ventricular), acute or chronic HF, and pericarditis.

### Statistical analysis

Continuous data are expressed as mean ± SD or median (IQR), depending on the normality of distribution. Categorical data are presented as counts with percentages (%). The independent *t*-test or Mann-Whitney *U* test was performed to assess differences in continuous variables between patients with and without the study outcomes. For differences in binary or categorical variables, the chi-squared test or Fisher exact test was used as appropriate. Cox proportional hazards (PH) regression analysis was conducted to evaluate the association between NT-proBNP and all-cause mortality. We evaluated whether the relationship between NT-proBNP and mortality was linear or nonlinear using restricted cubic splines within a Cox PH model. The nonlinear component of the spline was tested using a likelihood ratio test. Given the right-skewed distribution of NT-proBNP, we evaluated its association with mortality using both the original and log-transformed values. NT-proBNP showed a significant nonlinear association with mortality (*P* < 0.001). After log-transformation, the nonlinear component was no longer significant (*P* = 0.985). Therefore, NT-proBNP was log-transformed in the Cox regression models. HRs are presented along with 95% confidence intervals (CIs) in the format HR (95% CI). The PH assumption was evaluated using Schoenfeld residuals after fitting each univariable Cox regression model. Potential confounders of the association between NT-proBNP and mortality were identified using a directed acyclic graph, based on clinical knowledge. Kaplan-Meier survival curves were constructed for 5-year survival. Survival curves were compared using the log-rank test. A 2-sided *P* value of <0.05 was considered as statistically significant. All analyses were performed in R Version 4.3.1 (R Core Team 2023, R Foundation for Statistical Computing) and STATA version 17.0 (StataCorp 2021).

## Results

### Patient characteristics

**Figure 1:**
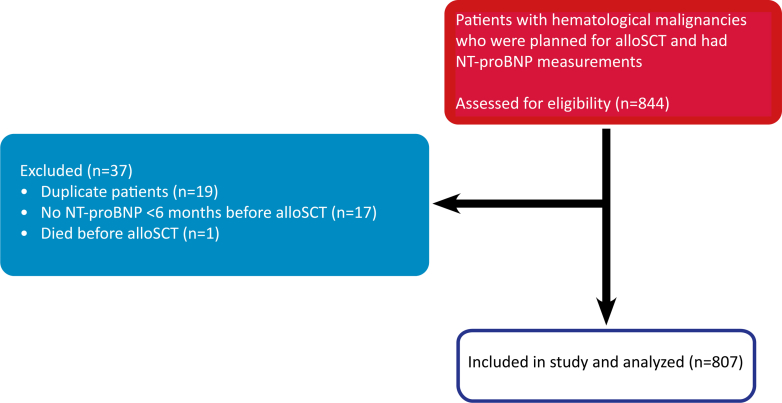
**STROBE Diagram** Figure 1 displays the process of patient inclusion for this retrospective cohort study. In total, 844 patients were screened for eligibility of whom 807 were included for analysis. alloSCT = allogeneic stem cell transplantation; NT-proBNP = N-terminal prohormone of brain natriuretic peptide; STROBE = Strengthening the Reporting of Observational studies in Epidemiology.

A total of 844 patients were screened for eligibility, of whom 807 met the inclusion criteria for analysis. The process of patient inclusion is displayed in Figure 1. Median age at alloSCT was 58.6 (47.9-66.0) years, and 278 patients (34.5%) were female. At baseline, 304 patients (37.7%) were prescribed cardiac medication <1 year before alloSCT, predominantly beta-blockers (n = 177), and 68 patients (8.4%) had cardiovascular risk factors or a history of CVD. The baseline characteristics of our study population are presented in Table 1. The most common cancer diagnosis was acute myeloid leukemia (n = 373), followed by non-Hodgkin lymphoma (n = 90) and acute lymphoblastic leukemia (n = 100). Median NT-proBNP at baseline was 113.2 (56.9-229.4) ng/L and baseline measurement was performed 25 (21-30) days before alloSCT. As conditioning therapy, 418 patients (51.8%) were treated with TBI with doses ranging from 2 to 12 Gray (Gy). Cyclophosphamide was administered in 461 patients (57.1%), fludarabine in 611 patients (75.7%), and busulfan in 373 patients (46.2%). Table 2 presents the oncological characteristics of our cohort.

**Table 1:** Baseline Characteristics

	Total (N = 807)	Alive (n = 414)	Died (n = 393)	*P* Value
Female, n (%)	278 (34.5%)	152 (36.7%)	126 (32.1%)	0.16
Age at alloSCT, y	58.6 [47.9-66.0]	56.3 [44.6-64.8]	61.0 [50.9-67.2]	<0.001
BMI, kg/m^2^ (n = 799)	25.0 [22.7-27.5]	24.8 [22.4-27.1]	25.3 [23.0-27.7]	0.02
Cardiac risk factors				
Any	68 (8.4%)	31 (7.5%)	37 (9.4%)	0.32
Hypertension	31 (3.8%)	14 (3.4%)	17 (4.3%)	0.49
Hypercholesterolemia	14 (1.7%)	3 (0.7%)	11 (2.8%)	0.030
Diabetes mellitus	19 (2.4%)	8 (1.9%)	11 (2.8%)	0.42
Smoking	20 (2.5%)	13 (3.1%)	7 (1.8%)	0.21
Cardiovascular history				
ACS	17 (2.1%)	9 (2.2%)	8 (2.0%)	0.89
Congestive heart failure	8 (1.0%)	4 (1.0%)	4 (1.0%)	1.00
Positive family history	8 (1.0%)	7 (1.7%)	1 (0.3%)	0.07
PM or ICD	5 (0.6%)	3 (0.7%)	2 (0.5%)	1.00
Stable angina pectoris	16 (2.0%)	8 (1.9%)	8 (2.0%)	0.92
(Supra)ventricular arrhythmias	33 (4.1%)	15 (3.6%)	18 (4.6%)	0.49
Valve abnormalities	4 (0.5%)	2 (0.5%)	2 (0.5%)	1.00
Pericarditis	2 (0.2%)	2 (0.5%)	0 (0.0%)	0.50
Congenital heart disease	1 (0.1%)	1 (0.2%)	0 (0.0%)	1.00
CABG	5 (0.6%)	3 (0.7%)	2 (0.5%)	1.00
Cardiac medication				
Any	304 (37.7%)	124 (30.0%)	180 (45.8%)	<0.001
Beta-blocker	177 (21.9%)	71 (17.1%)	106 (27.0%)	<0.001
Ascal	39 (4.8%)	19 (4.6%)	20 (5.1%)	0.74
P2Y12 inhibitor	38 (4.7%)	14 (3.4%)	24 (6.1%)	0.068
ACE inhibitor	83 (10.3%)	35 (8.5%)	48 (12.2%)	0.079
ARB	39 (4.8%)	17 (4.1%)	22 (5.6%)	0.32
Coumarines	76 (9.4%)	36 (8.7%)	40 (10.2%)	0.47
Cholesterol-lowering medication	94 (11.6%)	38 (9.2%)	56 (14.2%)	0.025
Laboratory values				
Hb, mmol/L	6.1 [5.2-6.8]	6.2 [5.3-7.0]	5.9 [5.2-6.6]	0.002
Leukocytes, x 10^9^/L	1.0 [0.3-2.4]	1.2 [0.4-2.8]	0.8 [0.2-2.0]	<0.001
CRP, mg/L (n = 405)	12.2 [5.0-45.7]	9.9 [4.2-33.4]	18.5 [6.0-63.6]	<0.001
Creatinine, µmol/L	60.0 [51.0-71.0]	58.0 [50.0-68.0]	62.0 [53.0-73.0]	<0.001
LDH, U/L	179.0 [153.0-213.0]	173.0 [149.0-205.0]	185.0 [157.0-223.0]	<0.001
Total cholesterol, mmol/L (n = 131)	4.4 [3.6-5.4]	4.6 [3.6-5.5]	4.4 [3.7-5.3]	0.92
LDL-cholesterol, mmol/L (n = 94)	2.5 [1.9-3.4]	2.5 [1.8-3.4]	2.5 [1.9-3.1]	0.95
HDL-cholesterol, mmol/L (n = 112)	1.1 [0.8-1.3]	1.1 [0.8-1.3]	1.1 [0.9-1.3]	0.96
Triglycerides, mmol/L (n = 125)	1.6 [1.0-2.3]	1.7 [1.2-2.3]	1.5 [0.9-2.3]	0.18
NT-proBNP, ng/L	113.2 [56.9-229.4]	100.2 [51.2-214.2]	122.4 [66.4-273.6]	0.001
NT-proBNP ≥125 ng/L	373 (46.2%)	179 (43.2%)	194 (49.4%)	0.08

ACE = angiotensin-converting enzyme; ACS = acute coronary syndrome; AlloSCT = allogeneic stem cell transplantation; ARB = angiotensin receptor blocker; BMI = body mass index; CABG = coronary artery bypass graft surgery; CRP = C-reactive protein; Hb = hemoglobin; HDL = high-density lipoprotein; ICD = implantable cardioverter-defibrillator; LDH = lactate dehydrogenase; LDL = low-density lipoprotein; NT-proBNP = N-terminal prohormone of brain natriuretic peptide; PM = pacemaker.

**Table 2:** Oncological Characteristics

	Total (N = 807)	Alive (n = 414)	Died (n = 393)	*P* Value
Type of cancer				0.001
AML	373 (46.2%)	204 (49.3%)	169 (43.0%)	0.07
ALL	100 (12.4%)	60 (14.5%)	40 (10.2%)	0.06
MDS	82 (10.3%)	46 (11.1%)	36 (9.2%)	0.36
MM/PCL	62 (7.7%)	17 (4.1%)	45 (11.5%)	<0.001
T-NHL	51 (6.3%)	26 (6.3%)	25 (6.4%)	0.96
B-NHL	39 (4.8%)	18 (4.3%)	21 (5.3%)	0.51
PMF	31 (3.8%)	15 (3.6%)	16 (4.1%)	0.74
MDS/MPD	23 (2.9%)	10 (2.4%)	13 (3.3%)	0.45
HL	11 (1.4%)	3 (0.7%)	8 (2.0%)	0.13
CML	10 (1.2%)	5 (1.2%)	5 (1.3%)	0.93
CLL	10 (1.3%)	1 (0.2%)	9 (2.3%)	0.010
Other	15 (1.9%)	9 (2.2%)	6 (1.6%)	0.50
Conditioning regimen				
T-cell depletion with alemtuzumab or alemtuzumab-ATG				<0.001
Flu-Bu	303 (37.5%)	105 (25.4%)	198 (50.4%)	<0.001
Cy-TBI 9 Gy	176 (21.8%)	80 (19.3%)	98 (24.4%)	0.08
Flu-Bu-cytarabine-Amsa	28 (3.5%)	9 (2.2%)	19 (4.8%)	0.039
Bu-Cy	5 (0.6%)	1 (0.2%)	4 (1.0%)	0.21
T-cell depletion with post-transplant cyclophosphamide				
Flu-Cy-TBI 2 Gy	207 (25.7%)	159 (38.4%)	48 (12.2%)	<0.001
Thiotepa-Flu-Bu	37 (4.6%)	29 (7.0%)	8 (2.0%)	0.001
TBI 12 Gy	7 (0.9%)	6 (1.4%)	1 (0.3%)	0.12
No T-cell depletion				
Flu-Cy-TBI 4 Gy	36 (4.5%)	21 (5.1%)	15 (3.8%)	0.39
TLI-ATG	8 (1.0%)	4 (1.0%)	4 (1.0%)	1.00
Immunosuppressive therapy after SCT				<0.001
None	500 (62.0%)	187 (45.2%)	313 (79.6%)	<0.001
Tacrolimus-MMF	251 (31.3%)	194 (46.9%)	57 (14.5%)	<0.001
Cyclosporine-MMF	44 (5.5%)	25 (6.0%)	19 (4.8%)	0.45
Cyclosporine	12 (1.5%)	8 (1.9%)	4 (1.0%)	0.39
Donor				0.83
Matched related	209 (25.9%)	107 (25.8%)	102 (26.0%)	0.97
Matched unrelated	549 (68.0%)	280 (67.6%)	269 (68.4%)	0.80
Haplo-identical	13 (1.6%)	6 (1.4%)	7 (1.8%)	0.71
Cord	36 (4.5%)	21 (5.1%)	15 (3.8%)	0.39
Conditioning therapy				
Alemtuzumab	508 (62.9%)	192 (46.4%)	316 (80.4%)	<0.001
Dose, mg	50 [20-50]	50 [20-50]	50 [20-50]	0.004
Amsacrine	28 (3.5%)	9 (2.2%)	19 (4.8%)	0.039
Dose, mg	794.0 [736.0-842.0]	756.0 [732.0-780.0]	812.0 [740.0-888.0]	0.06
Antithymocyte globulin	230 (28.5%)	68 (16.5%)	162 (41.2%)	<0.001
Dose, mg	75.0 [73.0-87.5.0]	75.0 [71.0-87.5]	75.5 [75.0-89.0]	0.12
Busulfan	373 (46.2%)	144 (34.8%)	229 (58.3%)	<0.001
Dose, mg	520.0 [448.0-600.0]	518.0 [456.0-680.0]	525.7 [448.0-592.0]	0.27
Cyclophosphamide	461 (57.1%)	290 (70.0%)	171 (43.5%)	<0.001
Dose, mg	6,000.0 [4,434.0-8,943.0]	7,000.0 [4,443.0-9,080.0]	5,460 [4,428.0-7,896.0]	0.017
Cytarabine	28 (3.5%)	9 (2.2%)	19 (4.8%)	0.039
Dose, mg	15,896.0 [14,754.0-16,874.0]	15,116.0 [14,672.0-15,760.0]	16,316.0 [14,836.0-17,760.0]	0.07
Fludarabine	611 (75.7%)	323 (78.0%)	288 (73.3%)	0.12
Dose, mg	300.0 [270.0-315.0]	300.0 [270.0-315.0]	300.0 [270.0-310.0]	0.93
Thiotepa	37 (4.6%)	29 (7.0%)	9 (2.0%)	<0.001
Dose, mg	820.0 [742.0-932.0]	792.0 [742.0-926.0]	896.0 [836.0-1061.0]	0.06
GVHD	381 (47.2%)	176 (42.5%)	205 (52.2%)	0.006
Stage GVHD				<0.001
1	97 (12.0%)	58 (14.0%)	39 (9.9%)	
2	102 (12.6%)	49 (11.8%)	53 (13.5%)	
3	75 (9.3%)	22 (5.3%)	53 (13.5%)	
4	29 (3.6%)	1 (0.2%)	28 (7.1%)	

TLI = total lymphoid irradiation; AML = acute myeloid leukemia; Amsa = amsacrine; ALL = acute lymphoblastic leukemia; ATG = antithymocyte globulin; B-NHL= B-cell non-Hodgkin lymphoma; Bu = busulfan; CML= chronic myeloid leukemia; CLL= chronic lymphocytic leukemia; Cord = cord blood transplantation (CBT); Cy = cyclophosphamide; Flu = fludarabine; GVHD = graft-versus-host disease; HL = Hodgkin lymphoma; MDS = myelodysplastic syndrome; MDS/MPD = myelodysplastic-myeloproliferative neoplasm; MMF = mycophenolate-mofetil; MM = multiple myeloma; NHL = T-cell non-Hodgkin lymphoma; PCL = plasma cell leukemia; TBI = total body irradiation; T-PMF = primary myelofibrosis.

### Follow-up and events

**Figure 2:**
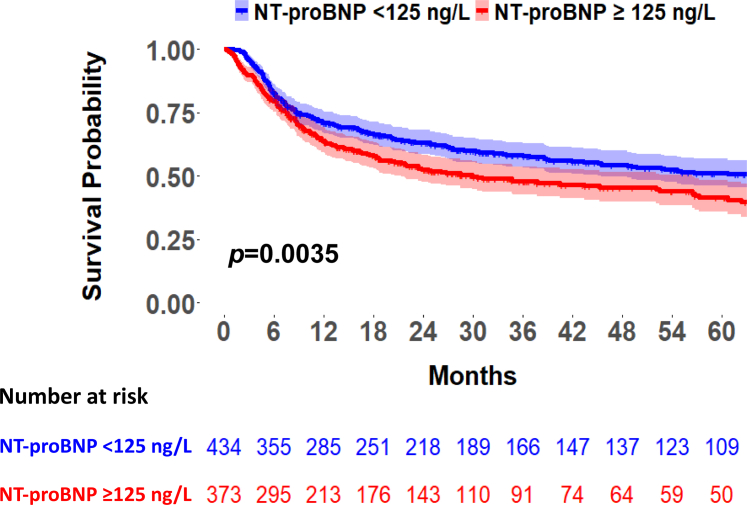
**Kaplan-Meier Curve for 5-Year Survival** This figure shows the 5-year survival after alloSCT for patients with a baseline NT-proBNP ≥125 ng/L compared to <125 ng/L. Patients with baseline NT-proBNP levels ≥125 ng/L have a significant increased risk of all-cause mortality (*P* = 0.0035). Abbreviations as in Figure 1.

**Figure 3:**
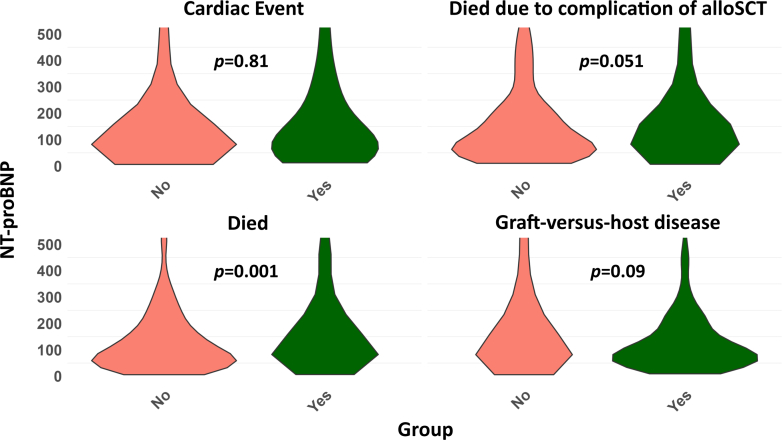
**Violin Plots of Baseline NT-proBNP for Different Outcomes** Violin plots for the difference in baseline NT-proBNP for the study outcomes cardiac events, all-cause mortality, death due to a complication of alloSCT and GVHD. Patients who died due to any cause of death allosct had a significant higher NT-proBNP at baseline. GVHD = graft-versus-host disease; other abbreviations as in Figure 1.

Patients were followed for a median period of 1.6 (0.6-4.0) years. During follow-up, 393 patients (48.7%) died, predominantly due to a complication of alloSCT including GVHD and autoimmune disease (61.8%) or tumor progression (36.6%). Only 6 patients (1.5%) had a primary cardiac cause of death. Patients who died had a significant higher baseline NT-proBNP compared to survivors (122.4 [66.4-273.6] ng/L vs 100.2 [51.2-214.2] ng/L, *P* = 0.001). Kaplan-Meier curves for 5-year survival showed a significant difference in survival for patients with a baseline NT-proBNP of ≥125 ng/L compared to <125 ng/L (*P* = 0.0035) (Figure 2, Central Illustration). The cause of death was divided into complications of alloSCT (GVHD, neutropenic sepsis, and autoimmune disease) and other causes of death. Patients who died due to a complication of alloSCT had a higher NT-proBNP at baseline compared to patients who died due to another cause of death (131.7 [75.0-299.1] ng/L vs 115.4 [53.6-228.5] ng/L; *P* = 0.051). Among 807 patients with a total follow-up of 2,234.6 patient-years, 76 cardiovascular events occurred, corresponding to an overall incidence rate of 3.4 events per 100 patient-years. During follow-up, 56 patients (6.9%) presented with one or more cardiac events of whom the majority with atrial or ventricular arrhythmias (n = 23), HF (n = 18), pericarditis (n = 16), and ACS (n = 12). No significant differences in baseline NT-proBNP were observed between patients who had a cardiac event compared to patients who did not experience this (*P* = 0.81). Univariable Cox PH regression analyses were conducted to evaluate the association between NT-proBNP ≥125 ng/L and each individual cardiovascular outcome, if the number of events was sufficient. We observed HRs of 1.64 (0.59-4.61) (*P* = 0.35) for HF, 0.80 (0.24-2.68) (*P* = 0.72) for ACS, 0.79 (0.31-2.0) (*P* = 0.62) for arrhythmias, 1.48 (0.40-5.53) (*P* = 0.56) for pericarditis, and 2.00 (0.13-32.0) for valvular disease. GVHD occurred in 381 patients (47.2%), of whom 97 (32.0%) had stage I, 102 (33.7%) stage II, 75 (24.8%) stage III, and 29 (9.6%) stage IV. Patients who developed any stage of GVHD had a significant higher risk of death compared to patients without GVHD: 52.2 vs 47.8%; *P* = 0.006. Figure 3 presents violin plots for the differences in baseline NT-proBNP for different study outcomes.

**Central Illustration:**
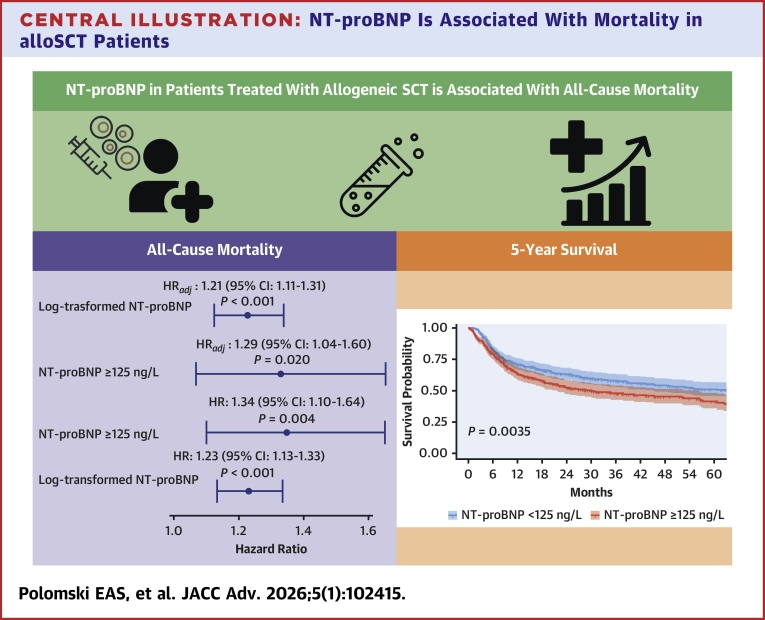
**NT-proBNP Is Associated With Mortality in alloSCT Patients** Baseline NT-proBNP is associated with increased risk of all-cause mortality with unadjusted HRs of 1.23 (1.13-1.33) (*P* < 0.001) and 1.34 (1.10-1.64) for NT-proBNP ≥125 ng/L (*P* = 0.004) and when adjusted for potential confounders HRs of 1.21 (1.11-1.31), (*P* < 0.001) and 1.29 (1.04-1.60), (*P* = 0.020) respectively. Moreover, 5-year survival was significantly lower in patients with NT-proBNP levels ≥125 ng/L compared to patients with levels <125 ng/L (*P* = 0.035). Abbreviations as in Figures 1 and 4.

### Association between NT-proBNP and all-cause mortality

**Figure 4:**
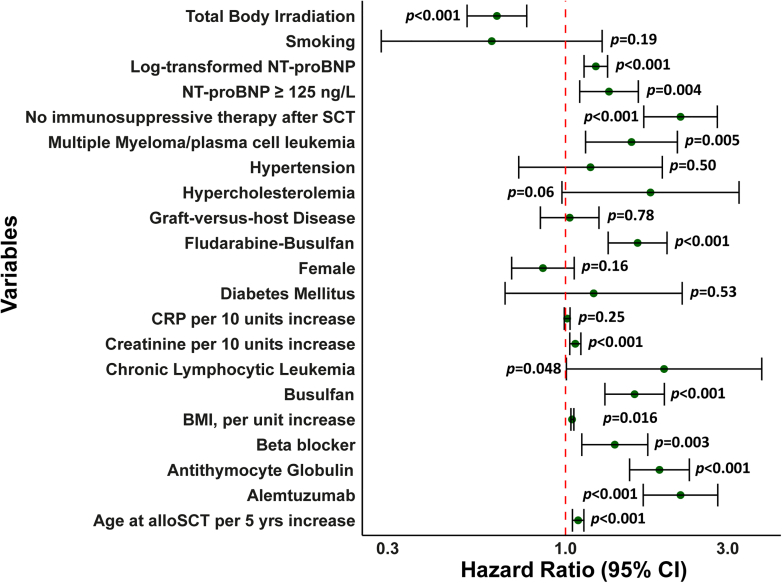
**Forest Plot of Univariable HRs for All-Cause mortality** Figure 4 displays the univariable hazard ratios for baseline variables on all-cause mortality. Log-transformed NT-proBNP, NT-proBNP ≥125 ng/L, diagnosis with multiple myeloma/plasma cell leukemia or chronic lymphocytic lymphoma, beta blocker use, baseline creatinine, conditioning treatment with fludarabine-busulfan, busulfan, alemtuzumab or antithymocyte globulin or no immunosuppressive therapy after alloSCT were positively associated with an increased risk of all-cause mortality. BMI = body mass index; SCT = stem cell transplantation; CRP = C-reactive protein; other abbreviations as in Figure 1.

Univariable Cox PH regression was performed to assess the association between baseline NT-proBNP and death, and the HRs were obtained. In the univariable model (Table 3, Figure 4), age at alloSCT per 5-year increase (HR: 1.09 [95% CI: 1.05-1.13], *P* < 0.001), log-transformed baseline NT-proBNP (HR: 1.23 [95% CI: 1.13-1.33], *P* < 0.001), and NT-proBNP ≥125 ng/L (HR: 1.34 [95% CI: 1.10-1.64], *P* = 0.004) were associated with an increased risk of mortality. In the multivariable model, corrected for sex and age at alloSCT, log-transformed NT-proBNP was positively associated with an increased risk of mortality (HR: 1.19 [95% CI: 1.09-1.29], *P* < 0.001). Confounders were identified using a directed acyclic graph. When the model was adjusted for all confounders (age at alloSCT, sex, body mass index, hypertension, smoking status, diabetes, history of ACS, creatinine, cancer diagnosis, and type of conditioning regimen), log-transformed NT-proBNP (HR: 1.21 [95% CI: 1.11-1.31], *P* < 0.001) and NT-proBNP ≥125 ng/L (HR: 1.29 [95% CI: 1.04-1.60] *P* = 0.020) were positively associated with an increased risk of mortality (Central Illustration).

**Table 3:** HRs Cox Proportional Hazards Model All-Cause Mortality

	HR [95% CI]	*P* Value	HR_adj_	*P* Value
Age at alloSCT, per 5 y increase	1.09 (1.05-1.13)	<0.001		
Female	0.86 (0.69-1.06)	0.16		
BMI, per unit increase	1.03 (1.01-1.06)	0.016		
Hypertension	1.18 (0.73-1.93)	0.50		
Hypercholesterolemia	1.78 (0.98-3.24)	0.06		
Diabetes	1.21 (0.66-2.20)	0.53		
Smoking	0.61 (0.29-1.28)	0.19		
Beta-blocker	1.40 (1.12-1.75)	0.003		
Creatinine, µmol/L	1.007 (1.003-1.01)	0.002		
CRP, mg/L	1.001 (0.999-1.003)	0.27		
MM/PCL	1.56 (1.15-2.13)	0.005		
CLL	1.95 (1.01-3.78)	0.048		
Busulfan	1.60 (1.31-1.95)	<0.001		
Alemtuzumab	2.18 (1.69-2.80)	<0.001		
Antithymocyte globulin	1.89 (1.54-2.31)	<0.001		
Fludarabine-busulfan	1.64 (1.34-1.99)	<0.001		
TBI	0.63 (0.77-0.80)	0.001		
No immunosuppressive therapy after SCT	2.18 (1.70-2.78)	0.001		
GVHD	1.03 (0.84-1.25)	0.78		
Log-transformed baseline NT-proBNP	1.23 (1.13-1.33)	<0.001	1.21 (1.11-1.31)	<0.001
NT-proBNP ≥125 ng/L	1.34 (1.10-1.64)	0.004	1.29 (1.04-1.60)	0.020

In the multivariable analysis, HR_adj_ is adjusted for confounders identified by a directed acyclic graph.

Abbreviations as in Tables 1 and 2.

## Discussion

Our results demonstrate the prognostic value of NT-proBNP in patients treated with alloSCT. NT-proBNP levels ≥125 ng/L at baseline are associated with a significant lower 5-year survival. Moreover, a significant higher baseline NT-proBNP is observed in patients who died due to any cause of death. This implicates the importance of NT-proBNP as a biomarker for (fragile) patients who are at risk for adverse events.

### Association between NT-proBNP and mortality

BNP is released into the circulation when cardiac wall stress increases and BNP as well as NT-proBNP are established markers in HF. In large cohorts of patients without HF, BNP has been found to be a stronger predictor of mortality compared to variables including estimated Glomerular Filtration Rate, chronic kidney disease, and body mass index. Also, strong associations between NT-proBNP and increased risk of cardiac events have been reported.^12^^,^^13^ In a cohort of multiple myeloma patients, NT-proBNP ≥300 ng/L was an indicator for overall survival and the authors suggested to incorporate NT-proBNP in determining the frailty of patients diagnosed with multiple myeloma.^14^ Elevated levels of NPs in patients without HF may result from other underlying conditions, such as vascular aging, subclinical cardiac disease, and poor cardiac reserve, and can therefore predict poor outcomes in acute and chronic conditions.^15^

### Prognostic value of NT-proBNP in patients after SCT

Cardiac biomarkers may detect early cardiovascular complications after SCT. Roziakova et al observed an increase in NT-proBNP and high-sensitive cardiac troponin T more than 2 weeks after alloSCT in one-third of the patients. Auberle et al^16^ found a linear pattern in the cumulative incidence of NT-proBNP in patients after alloSCT, possibly suggesting that NT-proBNP continues to increase in the long term.^17^ Therefore, measuring BNP before and during cancer treatment could provide valuable prognostic information.

Studies assessing the association between NT-proBNP before SCT and overall survival are scarce. One study conducted on 174 patients treated with alloSCT showed that higher levels of baseline NT-proBNP were associated with significant poorer outcomes regarding overall survival and increased risk of nonrelapse mortality.^18^ In a cohort of pediatric patients, elevated levels of BNP before SCT were associated with increased critical care utilization and higher mortality rates. To our knowledge, our study is the largest assessing the association between baseline NT-proBNP and mortality in adult patients undergoing alloSCT. As NPs have been shown to be of prognostic value in different populations, this underlines the importance of incorporating measurement of NPs in patients undergoing cancer treatment as this might identify frail patients at risk of adverse events such as GVHD, neutropenic sepsis, and autoimmune disease.

### Association between conditioning regimens, NT-proBNP, late cardiotoxicity, and mortality

Conditioning regimens with high-dose melphalan (>140 mg/m^2^) increase the risk of atrial fibrillation and arrhythmias that can again increase the risk of HF, especially in the presence of other (pre-existing) risk factors. Late cardiotoxicity after SCT has been well defined in prior research and the likelihood of its occurrence depends on comorbidities, GVHD, chemotherapy regimen, anthracycline dose, and radiation therapy.^7^^,^^19^

The most intense conditioning regimen for alloSCT is myeloablative conditioning. In general, patients treated with myeloablative conditioning are younger and have less comorbidities. In fragile patients or the presence of comorbidities like HF, switching to nonmyeloablative conditioning is considered. Currently, NT-proBNP is considered as a marker to refer patients for cardiac assessment before alloSCT, however, evidence is scarce and more research is needed including defining a cutoff value for NT-proBNP for cardiology referral. In our data, patients with NT-proBNP <125 ng/L were more often treated with myeloablative conditioning regimens (eg, TBI 9 Gy and cyclophosphamide) compared to nonmyeloablative regimens for patients with NT-proBNP ≥125 ng/L.

Our results showed that post-transplant cyclophosphamide was associated with a decreased risk of mortality and although our results suggest that TBI also seems to be associated with lower risk of mortality, we believe this is mostly driven by the regimens TBI was combined with, as only the combination of TBI 2 Gy with fludarabine and cyclophosphamide was associated with a decreased risk of mortality. Fludarabine was not associated with mortality in the univariable analysis, but we observed a significant decreased risk of mortality for patients that were administered cyclophosphamide. Therefore, the association between TBI and mortality is most likely driven by treatment with cyclophosphamide, as was also observed in prior research.^20^

After SCT, patients have a more than 7-fold increased risk of developing cardiovascular risk factors at a younger age compared to the general population, including hypertension, diabetes mellitus, and dyslipidemia.^21^ One study observed adverse cardiovascular events >1 year after alloSCT in 25% of the patients, mostly driven by changes in markers for HF (NT-proBNP >500 ng/L and reduced left ventricular ejection fraction <45%), which were not included in our composite endpoint of cardiovascular events. However, their incidence for atrial and ventricular arrhythmias and percutaneous coronary intervention was, respectively, 8% and 2%, which aligns with our findings.^16^ Higher risk of HF is considered a long-term side effect of alloSCT and Vasbinder et al reported a 6-month and 5-year incidence of 1.1% and 5 to 6%, respectively. New-onset HF was diagnosed in 0.4 to 2.2% of the patients^3^^,^^6^^,^^7^^,^^22^^,^^23^ following SCT. Although HF is generally diagnosed within 4 years after SCT, elevated NT-proBNP can already be seen in patients with (symptoms of) HF.^24^^,^^25^

Assessing the association between conditioning regimens, NT-proBNP, cardiotoxicity, and mortality could provide prognostic information for frail patients and future research should focus on tailored management of frail patients.

### Monitoring of patients with SCT

The ESC guidelines^9^ recommend cardiovascular assessment, electrocardiogram, and echocardiography before start of SCT in low-risk as well as high-risk patients. However, measuring NT-proBNP at baseline is currently a IIA recommendation. In our view, considering the prognostic value of NPs on mortality in different study populations and our results showing a positive association between baseline NT-proBNP and all-cause mortality, measuring baseline NT-proBNP before SCT should be considered. Studies have also shown an association between baseline troponin T and mortality in cancer^26^ and a study in patients undergoing SCT observed persistent elevation of troponin T up to 30 days after transplantation and presence of elevated troponin T before cardiac symptoms occurred.^24^ Therefore, not only NT-proBNP but also troponin T might be a useful biomarker to identify patients at risk of adverse (cardiac) events after SCT and more research should be performed on incorporating these cardiac biomarkers into risk prediction models. Risk prediction models can support the choice for patient-tailored treatment and depending on the risk scores, patient-specific monitoring and follow-up including routinely performing cardiac imaging and measuring cardiac biomarkers can be determined.^2^^,^^8^ Elevated NT-proBNP could possible identify patients at high-risk for mortality and adverse events who may undergo additional evaluation with echocardiography before alloSCT or less intense conditioning regiments may be considered. However, future studies should focus on the predictive value of echocardiography vs NT-proBNP to assess its correlation.

### Study Limitations

Troponin T levels were not routinely measured before alloSCT and, therefore, we were unable to assess the association between this biomarker and the study outcomes. Also, the majority of our patients was referred to our center from various community hospitals across the country for treatment with alloSCT and, therefore, detailed information on prior cancer treatment and doses was not available. Due to the limited number of patients with a cardiac cause of death, the association between baseline NT-proBNP and cardiac death could not be investigated. Furthermore, the number of cardiovascular events was relatively low (n = 54), limiting the power to assess the association between NT-proBNP and cardiovascular events. Moreover, as patients in our cohort did not undergo echocardiography, the relation between NT-proBNP and cardiac function could not be determined.

## Conclusions

Baseline NT-proBNP in patients treated with alloSCT is an independent predictor of all-cause mortality. Measuring baseline NT-proBNP can identify high-risk patients and may improve the risk stratification of patients treated with alloSCT.

Perspectives **COMPETENCY IN MEDICAL KNOWLEDGE:** NT-proBNP in patients treated with allogeneic SCT is associated with an increased risk of all-cause mortality. Moreover, patients with baseline NT-proBNP ≥125 ng/L, as defined by the guidelines, show increased risk of all-cause mortality.

**TRANSLATIONAL OUTLOOK:** Future research should explore the relation between baseline NT-proBNP and cardiotoxicity evaluated with echocardiography in patients undergoing alloSCT to gain more insight in the predictive value of cardiac biomarkers.

## Funding support and author disclosures

The authors have reported that they have no relationships relevant to the contents of this paper to disclose.

## Supplementary material

STROBE_checklist_cohort
